# Mass Cytometry reveals unique phenotypic patterns associated with subclonal diversity and outcomes in multiple myeloma

**DOI:** 10.1038/s41408-023-00851-5

**Published:** 2023-05-22

**Authors:** Linda B. Baughn, Erik Jessen, Neeraj Sharma, Hongwei Tang, James B. Smadbeck, Mark D. Long, Kathryn Pearce, Matthew Smith, Surendra Dasari, Zohar Sachs, Michael A. Linden, Joselle Cook, A. Keith Stewart, Marta Chesi, Amit Mitra, P. Leif Bergsagel, Brian Van Ness, Shaji K. Kumar

**Affiliations:** 1 Division of Laboratory Genetics, Department of Laboratory Medicine and Pathology, Mayo Clinic, Rochester, MN USA; 2 Division of Hematopathology, Department of Laboratory Medicine and Pathology, Mayo Clinic, Rochester, MN USA; 3 Division of Computational Biology, Department of Quantitative Health Sciences, Mayo Clinic, Rochester, MN USA; 4 Department of Biostatistics and Bioinformatics, Roswell Park Comprehensive Cancer Center, Buffalo, NY USA; 5 Division of Hematology, Department of Internal Medicine, Mayo Clinic, Rochester, MN USA; 6 Division of Hematology, Oncology, and Transplantation, Department of Medicine and Masonic Cancer Center, University of Minnesota, Minneapolis, MN USA; 7 Department of Laboratory Medicine and Pathology, University of Minnesota, Minneapolis, MN USA; 8 Princess Margaret Cancer Centre, Toronto, ON Canada; 9 Division of Hematology, Department of Internal Medicine, Mayo Clinic, Scottsdale, AZ USA; 10 Department of Drug Discovery and Development, Auburn University, Auburn, AL USA; 11 Department of Genetics, Cell Biology and Development, University of Minnesota, Minneapolis, MN USA

**Keywords:** Myeloma, Oncogenes

## Abstract

Multiple myeloma (MM) remains an incurable plasma cell (PC) malignancy. Although it is known that MM tumor cells display extensive intratumoral genetic heterogeneity, an integrated map of the tumor proteomic landscape has not been comprehensively evaluated. We evaluated 49 primary tumor samples from newly diagnosed or relapsed/refractory MM patients by mass cytometry (CyTOF) using 34 antibody targets to characterize the integrated landscape of single-cell cell surface and intracellular signaling proteins. We identified 13 phenotypic meta-clusters across all samples. The abundance of each phenotypic meta-cluster was compared to patient age, sex, treatment response, tumor genetic abnormalities and overall survival. Relative abundance of several of these phenotypic meta-clusters were associated with disease subtypes and clinical behavior. Increased abundance of phenotypic meta-cluster 1, characterized by elevated CD45 and reduced BCL-2 expression, was significantly associated with a favorable treatment response and improved overall survival independent of tumor genetic abnormalities or patient demographic variables. We validated this association using an unrelated gene expression dataset. This study represents the first, large-scale, single-cell protein atlas of primary MM tumors and demonstrates that subclonal protein profiling may be an important determinant of clinical behavior and outcome.

## Introduction

Multiple myeloma (MM) is the second most common hematopoietic malignancy [1, 2], characterized by a clonal expansion of malignant antibody-producing post-germinal-center plasma cells (PCs) within the bone marrow (BM). Although the overall survival of patients with MM has improved over time owing to the use of novel chemotherapeutic agents, targeted therapies, and autologous stem cell transplantation [3], MM remains an incurable malignancy in most patients [4, 5].

Bulk and single-cell studies using karyotype analysis, DNA sequencing methodologies, fluorescence in situ hybridization (FISH), and genomic microarrays have revealed extensive intratumoral genetic complexity and heterogeneity in MM [6–19]. Genetically distinct MM subclones evolve over time and contribute to chemotherapeutic resistance and disease progression. Recent studies using single-cell RNA sequencing have provided transcriptomic characterization of MM tumor heterogeneity [20–25], but protein-level characterization has not been well-reported [26]. Since most modern therapies target protein pathways, understanding proteomic heterogeneity may provide an opportunity for novel protein-based therapeutic interventions.

One methodology to assess single-cell proteomic heterogeneity includes mass cytometry (CyTOF) [27], which allows multiplex phenotyping of single cells providing the potential for novel protein signature identification that may be associated with chemotherapeutic resistance and/or disease outcome. CyTOF utilizes transition element isotype-tagged antibodies using time-of-flight mass spectrometry, allowing the detection of up to 40 to 50 simultaneous epitopes in single cells without significant spectral overlap. Previous studies using traditional flow cytometry have identified significant changes in the protein expression of numerous markers associated with MM disease survival, relapse, and/or drug resistance using this approach [28–30]. Here, we used CyTOF to analyze 34 epitopes in 49 primary MM patient samples at the single-cell level. We provide an integrated, comprehensive, single-cell atlas of cell surface molecules, transcription factors, and phosphoprotein targets that were previously individually associated with drug response or resistance in MM. We detect subclonal protein profiles that are shared among patients and associated with important clinical variables, including survival.

## Materials and methods

### Patient samples

All samples were referred to the Mayo Clinic Genomics Laboratory for routine clinical testing. Forty-nine cryopreserved BM samples from patients diagnosed with MM were selected for analysis by CyTOF based primarily on the abundance of PCs in the BM and having at least 500 live light-chain-restricted PCs. This retrospective study was approved by the institutional review board of the Mayo Clinic. A minimal risk waiver for consent was obtained for this study.

### Clinical data

FISH, S-phase calculations, and stratification for myeloma and risk-adapted therapy (mSMART, msmart.org) assessments were performed as part of routine clinical practice. FISH analysis of immunoglobulin (cIg)-stained positive PCs studies were performed as previously described [31] using probes described in Supplementary Materials and Methods. Plasma cell S-phase data were obtained from flow cytometry, as described in [32]. Each patient’s treatment response at 90 days was based on the International Myeloma Working Group uniform response [33] and further described in Supplementary Materials and Methods.

### CyTOF antibody panel

Thirty-seven antibody targets (Supplementary Table 1) directed against cell surface and intracellular proteins were initially designed using the web-based panel designer software Maxpar Panel Designer (Fluidigm/Standard BioTools, South San Francisco, CA) for optimal signals, minimum background due to oxidation, isotopic purity, and sufficient sensitivity for each targeted marker. Prelabelled antibodies were purchased from Fluidigm. Purified antibodies requiring conjugation were purchased from BioLegend (San Diego, CA) or Sigma Aldrich (St. Louis, MO) and labelled using the X8 polymer Maxpar antibody conjugation kit (Fluidigm) according to manufacturer’s instructions. Each antibody was validated and titrated against positive and negative control cell lines.

### Sample preparation for flow cytometry and CyTOF

Total BM cells were processed using ammonium, chloride, and potassium (ACK) lysis buffer (Thermo Fisher Scientific, Waltham, MA) to lyse red blood cells and centrifuged for 5 min at 1350 RPM. The pellet was washed with PBS, centrifuged for 5 min at 1350 RPM and resuspended in Chang BMC medium containing 20% fetal bovine serum (FBS) (Irvine Scientific, Santa Ana, CA). A portion of the cells was prepared for FISH as part of routine clinical testing. The remaining cells were centrifuged at 1200 rpm for 8 min and the cell pellets were resuspended in 1.5 ml of 10% DMSO (Sigma Aldrich) in Chang BMC medium, added to a cryotube, and frozen in liquid nitrogen.

Prior to CyTOF staining, three randomly selected BM samples from the liquid nitrogen biobank were thawed and stained by flow cytometry to evaluate the abundance of total viable PCs. Each cryovial was quickly thawed and the cells were immediately washed and centrifuged at 500 x g for 5 min in 20% FBS (Thermo Fisher Scientific, Waltham, MA, USA) in RPMI1640 and 1:10,000 benzonase nuclease (Sigma Aldrich). The cell pellets were resuspended in residual RPMI solution, washed again with the same medium and resuspended in cell staining buffer including 1x PBS, 3% BSA, and 0.1% sodium azide, followed by surface staining using the following antibodies: CD38 HIT2 (Cat# 303504), CD229 HLy-9.1.25 (Cat# 326108), CD138 MI15 (Cat# 356524), and CD45 HI30 (Cat# 304026) (BioLegend). Samples were processed using a BD Accuri C6 Plus Personal Flow Cytometer (BD Biosciences, San Jose, CA) (Supplementary Fig. 1A).

For CyTOF cell staining (reagents described in Supplementary Table 2), frozen BM cells were rapidly thawed and suspended in warmed RPMI1640 with 20% FBS containing 1:10,000 benzonase nuclease. Additional CyTOF staining details are described in Supplementary Materials and Methods. Briefly, 1–3 million cells were washed, incubated with Cell-ID and resuspended in 1x Maxpar Fix and Perm Buffer. Cells were subjected to the cell surface antibody panel, washed with Maxpar Cell Staining Buffer (CSB) and stored overnight at −80 °C. Cells were subjected to the intracellular antibody panel, fixed with a fresh 1.6% formaldehyde solution (Thermo Fisher Scientific), washed with CSB and resuspended in 1 ml of Cell-ID Intercalator-Lr solution. Samples were barcoded using the Cell-ID 20-Plex Pd Barcoding Kit and combined with the Maxpar cell acquisition solution and EQ four-element calibration beads followed by acquisition on the Helios CyTOF system. Data were collected as flow cytometry standard (FCS) files, debarcoded and normalized to the acquired calibration bead signal.

### Processing of Flow Cytometry Standard (FCS) files

The workflow of further processing of the FCS files is shown in Supplementary Fig. 1B and described in detail in Supplementary Materials and Methods. Briefly, normalized FCS files were analyzed by the Maxpar Pathsetter software (version 2.0; Fluidigm) for cleanup to select live cell events. To assess batch variability, unstimulated and cytokine-activated Veri-Cell reference standards (see Supplementary Materials and Methods) were compared between the batches. As expected [27], PMA and ionomycin stimulation resulted in consistent increases in pp38, pERK, pCREB, Ki-67, and pS6 and reductions in total IkBα (Supplementary Fig. 1C). The markers SOX2, CD27, and CD147 were subsequently removed from the downstream analysis because of batch variations among patient samples. Additional processing steps including cell modeling (Supplementary Figure 1D) including criteria for inclusion of MM cells (Supplementary Fig. 1E) and further data processing are described in Supplementary Materials and Methods.

### Comparison to clinical metrics

The log2 fold change of marker expression was calculated to identify differences in the average marker abundance, proportion of cells with marker changes from clusters, and proportion of cells assigned to meta-clusters for the 90 day treatment response (poor vs. good), sex (male vs. female), age ( > 60 vs. ≤ 60 years), type (relapsed refractory MM (RRMM) vs. newly diagnosed (NDMM), primary (hyperdiploidy vs. 11;14), *TP53* deletion (present vs. absent), 1q gain (present vs. absent), *MYC* disruption (present vs. absent), monosomy 13 (present vs. absent), deletion 13q (present vs. absent), mSMART (high vs. standard), and S-phase (high vs. low). Statistical significance was determined using an unpaired t-test (t-test of independent means) between values for each comparison. The correlation coefficients between a pair of binary variables were calculated with Cramer’s V method with signs (+/−) introduced from a Pearson’s correlation test, which was used to estimate all of the remaining correlations. Violin plots were generated using the GraphPad Prism software (San Diego, CA).

### Survival analysis

Overall survival (OS) was defined as the time from diagnosis to death from any cause or the last follow-up, with those alive censored at the date of the last follow-up. The APEX phase-3 clinical trial was used as a validation cohort. Pretreatment gene expression and clinical outcomes data on the APEX phase-3 clinical trial (039; *n* = 156) on relapsed and/or refractory myeloma patients were downloaded from the Gene expression omnibus (GEO) database (GSE9782) as Affymetrix HG-U133A/B gene probe set analysis dataset. The APEX data set (GSE9782; *n* = 264) contains gene expression data on phase-2 and phase-3 relapsed and/or refractory myeloma clinical trials, including APEX phase-3 trial (039), a APEX companion study (040), SUMMIT (025) and CREST phase-2 trials (024) [34]. Median gene intensity scores were calculated for the following genes that served as meta-cluster 1 gene signature: *BCL-2, IKZF3, MYC, NFKBIA (*IkBα*)*, and *PTPRC (CD45)*. A combined meta-cluster activity score was computed for each sample as the summation of the ascending ranks of expression of median gene intensities. Survival curves were estimated using Kaplan-Meier analysis and compared using the log-rank test. Cox regression analysis was performed to estimate the survival risk of the different phenotypic meta-clusters for overall survival. Statistical analyses were performed using BlueSky (Chicago, IL, USA), with a value of significance defined as *P* < 0.05.

## Results

### Description of patient cohort

A total of 49 BM samples from patients with MM were selected for CyTOF analysis (Table 1). The median age was 67 years (range 46–95 years), with a male predominance ( ~ 2:1 M:F ratio). Most samples (93.9%) had available flow cytometry data as part of routine clinical care. Of these samples, 59.2% had kappa restriction and the rest had lambda restriction. The median percentage of kappa- or lambda-restricted monotypic PCs was 31% of all nucleated cells and the percentage of polytypic PCs in relation to all PCs examined was ≤ 5% in 93.9% of samples. The median S-phase percentage of the monotypic PCs was 1.5% with 34.7% having ≥ 2% S-phase reflective of high-risk disease. Twenty samples (40.8%) were newly diagnosed MM (NDMM) and 29 (59.2%) were relapsed and/or refractory MM (RRMM). A primary, recurrent cytogenetic abnormality determined by FISH analysis was identified in 95.9% of samples, with most (79.6%) exhibiting either hyperdiploidy (49.0%) or a t(11;14) translocation (30.6%). The remaining samples had t(4;14) (8.2%), t(14;20) (4.1%), or hyperhaploidy (4.1%). Secondary cytogenetic abnormalities included *TP53* loss (24.5%), 1q gain or amplification (57.1%), *MYC* rearrangement (26.5%), and monosomy 13 or 13q deletion (69.4%). Most samples (69.4%) were high-risk using the Mayo Clinic mSMART risk stratification (www.msmart.org), and the remainder (30.6%) were standard risk. The depth of response following treatment was: VGPR (14.3%) PR, (40.8%) MR, (12.2%) SD, (14.3%); and PD (18.4%) (Table 1).

**Table 1:** Patient characteristics.

Characteristic (*N* = 49)	*N* (%)
**Age**	
Median	67 years
Range	46–95 years
**Sex**	
Male	33 (67.3)
Female	16 (32.7)
**Light chain**	
Kappa	29 (59.2)
Lambda	20 (40.8)
**Monotypic PC percentage by flow cytometry**	
Median	31%
Range	7–88%
7–19	12 (24.5)
20–39	21 (42.9)
40–59	5 (10.2)
60–79	6 (12.2)
80–99	2 (4.1)
No data	3 (6.1)
**Polytypic PC by flow cytometry**	
≤ 5%	37 (93.9)
No data	3 (6.1)
**S-phase by flow cytometry**	
Median	1.5%
Range	0.2–12%
0.2–0.9	12 (24.5)
1–1.9	13 (26.5)
2–5.9	8 (16.3)
6–12	9 (18.4)
No data	7 (14.3)
mSMART risk	
Standard	15 (30.6)
High	34 (69.4)
NDMM vs. F/U	
NDMM	20 (40.8)
Relapsed and/or refractory	29 (59.2)
Depth of response	
VGPR	7 (14.3)
PR	20 (40.8)
MR	6 (12.2)
SD	7 (14.3)
PD	9 (18.4)
**Primary cytogenetic abnormality**	
Hyperdiploidy	24 (49.0)
t(11;14)	15 (30.6)
t(4;14)	4 (8.2)
t(14;20)	2 (4.1)
Hyperhaploid	2 (4.1)
Undefined primary	2 (4.1)
**Secondary cytogenetic abnormality**	
*TP53* deletion	12 (24.5)
1q gain or amplification	28 (57.1)
*MYC* rearrangement	13 (26.5)
Monosomy 13 or 13q deletion	34 (69.4)

To evaluate whether integrated cell surface and signaling protein profiles can identify clinically significant subtypes of MM, we analyzed these 49 samples using a custom CyTOF panel targeting 34 cell surface or intracellular proteins simultaneously within single cells. The panel was designed to target protein markers associated with MM disease survival, disease relapse, or drug resistance (Supplementary Table 3). The targets included 7 phosphorylated proteins representing mitogenic signaling pathways (pAKT, pCREB, pERK, pp38, pRB, pS6, and pSTAT3), 5 myeloma-specific transcription factors (MYC, IkBα, IKZF1 (Ikaros), IKZF3 (Aiolos), and IRF4 (MUM-1)), 2 survival proteins (BCL-2 and MCL-1), a component of E3 ubiquitin ligase (CRBN, Cereblon) implicated in lenalidomide response, a marker of programmed cell death (cleaved caspase 3, clCasp3), a marker of proliferation (Ki-67), 15 cell surface markers (CD117, CD138, CD16, CD19, CD20, CD28, CD3, CD34, CD38, CD45, CD56, CD81, CD71, CD49d, and CD274), and intracellular kappa and lambda light chains. Monotypic PCs were defined as CD16^−^, CD3^−^, CD19^−^, CD138^+^, IRF4^+^, and kappa^+^ or lambda^+^, with light chain restriction for each patient, consistent with clinical flow cytometry data.

### CyTOF analysis recapitulates expected protein expression patterns in MM

**Fig. 1:**
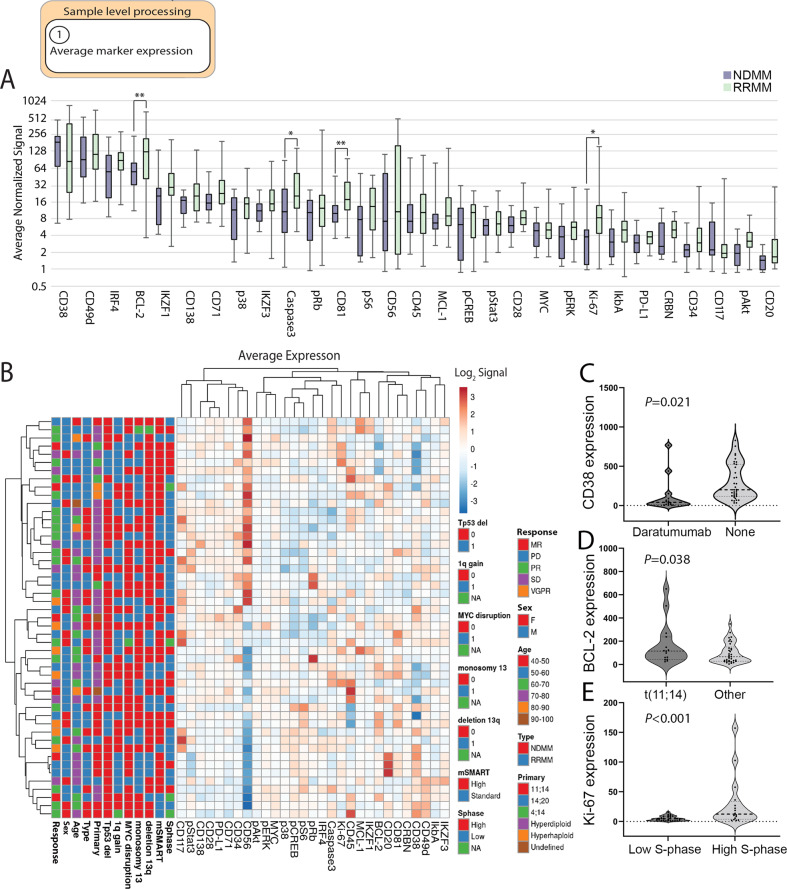
Sample level processing: Average marker expression and correlation with clinical metrics, patient demographics and tumor genetics. **A** Distribution of normalized marker signal in samples averaged across all cells according to NDMM (N) or RRMM (R) status. Markers with significant differences in bulk expression between NDMM and RRMM samples are indicated with a *P-values: ** < 0.01, *< 0.05. **B** Clustering of average, normalized marker values with patient clinical metrics, demographics and tumor genetics. Signal is displayed in log_2_. Cohort metrics defining response to treatment, sex, age, type (NDMM or RRMM), primary genetic abnormality, *TP53* deletion, *MYC* disruption, monosomy of 13, deletion 13q, mSMART score, and S-phase value. **C** Violin blots displaying the CD38 expression in patients that had been exposed to daratumumab within 6 months prior to sample collection compared to patients without daratumumab treatment within 6 months prior to sample collection (None). **D** Expression of BCL-2 in patients with t(11;14) compared to non-t(11;14) (Other). **E** Expression of Ki-67 in patients with low S-phase compared to high S-phase.

First, we analyzed our data and compared our findings to previously published data in MM. We compared the average normalized signal of each marker within each patient’s bulk PC population across the dataset. Among the cell surface proteins, CD38, CD49d, CD138, and CD71 were the most abundant, while CD20, a late-stage B cell marker, was the least abundant, as previously reported [35] (Fig. 1A). Expression of CD56, a transmembrane glycoprotein known to be aberrantly expressed in MM [36], was present in 24 (49.0%) and absent in 25 (51.0%) samples (Fig. 1A, B). Consistent with previous reports [37, 38], the average surface expression of the PC marker CD138 was lower than that of CD38; cryopreservation and sample processing have been reported to reduce CD138 surface expression. In addition, detection of surface CD38 protein is influenced by treatment with the CD38-targeting antibody daratumumab [39], interfering with CD38 identification 4-6 months after the last exposure [40–42]. Accordingly, the fourteen samples (28.6%) from patients who had been exposed to daratumumab within 6 months prior to collection displayed reduced CD38 abundance (treatment: 40.5, range 4.1–767.3 vs. none: 201.7, range 33.2–829.0, *P* = 0.021, Fig. 1C, Supplementary Table 4).

Among the intracellular proteins, transcription factors IRF4 and IKZF1 were among the most abundant, as recently reported [43] (Fig. 1A). Higher levels of BCL-2 relative to MCL-1 have been reported in association with t(11;14) MM; a feature associated with increased sensitivity to the BCL-2 inhibitor venetoclax [44, 45]. Consistent with those studies, samples with t(11;14) had a higher BCL-2/MCL-1 expression ratio and higher median BCL-2 levels than the non-t(11;14) samples (BCL-2/MCL-1 ratio: 11.8, range 0.5–91.1 vs. 6.3, range 0.3–55.1, *P* = 0.057; median BCL-2 expression: 114.7, range 24.3–650.9 vs. 66.0, range 3.6–348.7, *P* = 0.038) (Fig. 1D). In contrast, the median expression of MCL-1 was similar between the two groups (8.6, range 3.0–79.0 vs. 8.1, range 2.5–144.2, *P* = 0.228) consistent with the essential role for MCL-1 in MM survival. As expected [46], cases with high S-phase ( ≥ 2%), had a greater median expression of Ki-67 than cases with low S-phase ( < 2%) (12.3, range 1.7–157.3 vs. 4.4, range 1.0–15.4, *P* < 0.001) (Fig. 1E). In addition, a significant positive correlation was identified between expression of Ki-67, MYC and pERK, between pAKT and MYC and between MCL-1 and IKZF1 (Supplementary Figure 2).

In addition, we identified numerous proteins with higher abundance in RRMM compared to NDMM samples (Fig. 1B). The expression levels of BCL-2 (*P* < 0.01), clCasp3 (*P* = 0.04), Ki-67 (*P* = 0.04) and CD81 which has been associated with chemoresistant MM tumor cells [47] (*P* < 0.01), were significantly higher in RRMM than in NDMM samples (Fig. 1B) consistent with a more aggressive disease. These data using bulk protein expression demonstrate that CyTOF profiling recapitulates key protein features of MM as previously reported.

### Thirteen commonly expressed phenotypic meta-clusters can be detected among patients

**Fig. 2:**
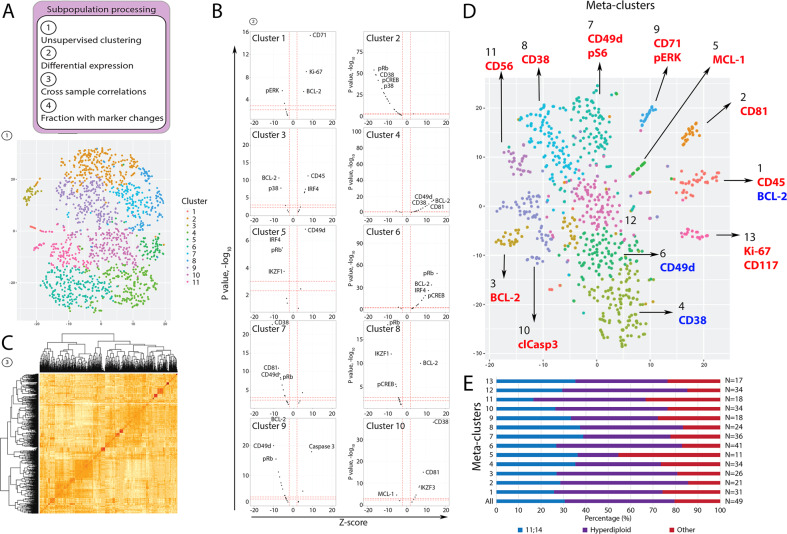
Subpopulation processing: Unsupervised clustering approach resulting in identification of 13 phenotypic meta-clusters across all patient samples. **A** Top: Overall schematic of the subpopulation processing from Supplementary Fig. 1. Bottom: Unsupervised clustering of a single patient’s sample. Cells are colored based on the cluster assigned. **B** Differential protein expression results from MAST output showing driving proteins for each cluster. Red vertical lines denote a Z-score greater than or less than 1. Red horizontal lines represent a *p*-value of 0.001 and 0.005. The top hits for each cluster are labelled. **C** Correlation plot of all Z-scores from the MAST output for all clusters from all patients (1400). **D** t-SNE of all unsupervised clusters between samples colored by the meta-cluster assignment with labelling of the significant markers in each meta-cluster with CD38 included. The top significant markers with either relative increased (red) or decreased (blue) changes are listed by the meta-cluster with the most significant meta-cluster differentiating marker bolded. **E** Distribution of primary cytogenetic abnormality in each meta-cluster (blue t(11;14), purple (hyperdiploidy), red (other including hyperhaploidy, t(4;14), t(14;20) and undefined). Number on the right indicate the number of cases with evidence of each phenotypic meta-cluster.

Next, we determined whether our integrated cell surface and intracellular protein dataset could be used to detect clinically meaningful patterns of protein expression among subpopulations of MM tumors. First, to identify common phenotypic meta-clusters among our patient cohort, we used an unsupervised clustering analysis to define phenotypic clusters in each patient sample individually (Fig. 2A and Supplementary Materials and Methods). This analysis revealed 11–29 phenotypic clusters per patient. A representative sample of RRMM with t(11;14) and *TP53* deletion is shown in Fig. 2A.

To define the phenotypic clusters further, we next performed a differential abundance analysis to identify significant markers that differed in their expression between clusters in each patient (Fig. 2B). This analysis generated Z-scores for each marker showing the degree of directionality of the change in expression (increased expression, positive Z-scores and decreased in expression, negative Z-scores). Next, to determine if phenotypic clusters were shared across patients, individual patient clusters were divided into meta-clusters using the Z-score data as inputs for unsupervised clustering to identify cluster-matched equivalents among all patients (Fig. 2C). This allowed us to characterize similar phenotypic clusters across all patients revealing 13 phenotypic meta-clusters among the 49 MM samples (Fig. 2D). Each phenotypic meta-cluster was defined by the most highly differentially expressed protein markers (absolute Z-score of > 5 and significant changes in > 50% of cells within the meta-cluster).

The phenotypic meta-clusters were as follows: Meta-cluster 1 (CD45hi, BCL-2lo), Meta-cluster 2 (CD81hi), Meta-cluster 3 (BCL-2hi), Meta-cluster 4 (CD38lo), Meta-cluster 5 (MCL-1hi), Meta-cluster 6 (CD49dlo), Meta-cluster 7 (CD49dhi and pS6hi), Meta-cluster 8 (CD38hi), Meta-cluster 9 (CD71hi and pERKhi), Meta-cluster 10 (clCasp3hi), Meta-cluster 11 (CD56hi), and Meta-cluster 13 (Ki-67hi and CD117hi) (Fig. 2D, Supplementary Table 5). Group 12 showed no significant change in the expression of any marker. Each phenotypic meta-cluster was shared by at least 11 patient samples. Phenotypic meta-cluster 6 was present in the greatest number (*n* = 41) of patient samples, while meta-cluster 5 was present in the least number of patient samples (*n* = 11). The fraction of cells in each of the 49 patient samples belonging to each of the 13 phenotypic meta-clusters and the fraction of cells in each phenotypic meta-cluster belonging to each of the 49 patient samples is shown in Supplementary Fig. 3. Except for meta-clusters 5 and 11, the overall distribution of primary cytogenetic abnormalities was similar across the phenotypic meta-clusters (Fig. 2E).

### Phenotypic meta-cluster composition is correlated with clinical behavior

**Fig. 3:**
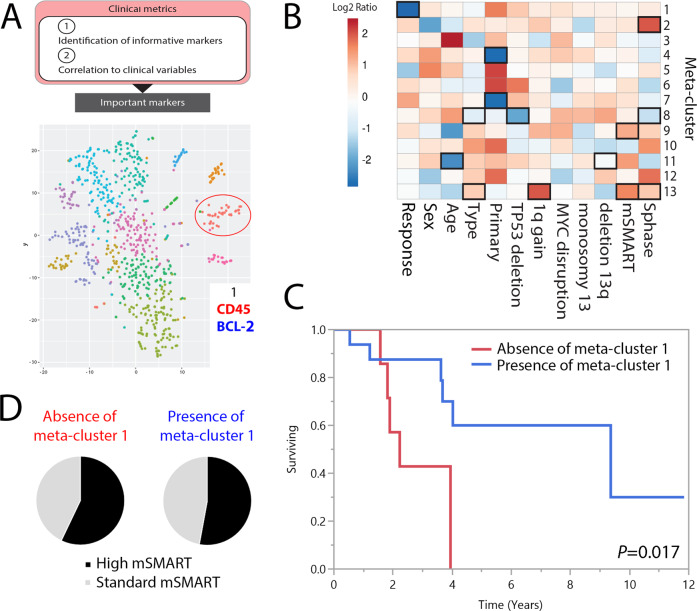
Comparison of the 13 phenotypic meta-clusters with clinical metrics, patient demographics, tumor genetics and disease outcome. **A** Overall schematic of workflow from subpopulation processing to clinical metric correlation. Meta-cluster 1 (red circle) was the only subpopulation associated with a significant difference in response to therapy and disease outcome. **B** Heatmap showing the log2 fold change in the proportion of cells within a sample belonging to each meta-cluster for different clinical and cohort metrics. Significant (*P* < 0.05) changes are highlighted in a black box. Cohort metrics defining response to treatment (poor including PD vs. good including PR and VGPR), sex (male vs. female), age (> 60 vs. ≤ 60 years), type (RRMM vs. NDMM)), primary genetic abnormality (hyperdiploidy vs. 11;14), *TP53* deletion (present vs. absent), *MYC* disruption (present vs. absent), monosomy 13 (present vs. absent), deletion 13q (present vs. absent), mSMART score (high vs. standard), and S-phase value (high vs. low). **C** A comparison of OS (years) in patients with absence of meta-cluster 1 (red line) and in patients with presence of meta-cluster 1 (blue line). OS time (median) was 2.2 [95%CI: 1.6–3.9] years (*n* = 7) and 9.4 [95%CI: 3.6-not calculated] years (*N* = 17) in the 2 meta-clusters, respectively. **D** Distribution of high and standard mSMART (*TP53* deletion and/or 1q gain) among the 7 patients without meta-cluster 1 and 17 patients with meta-cluster 1.

Next, we asked whether the proportion of individual phenotypic meta-clusters within each patient correlated with clinical variables including tumor genetics, response to therapy and disease outcome (Fig. 3A). The fold change in the proportion of cells within a sample belonging to each meta-cluster is indicated in the heatmap and correlated to clinical variables (Fig. 3B). Tumors associated with a poor depth of response had a significantly reduced abundance of meta-cluster 1, which was characterized by elevated CD45 and low survival protein BCL-2 along with elevated IKZF3, MYC and IkBα, suggesting that phenotypic meta-cluster 1 may be responsive to MM therapy. Meta-cluster 13, characterized by increased expression of the proliferative marker Ki-67 and CD117 as well as the IKZF1 transcription factor and pRB (Fig. 2D, Supplementary Table 5) was significantly more prevalent in RRMM samples and in those with 1q gain, elevated mSMART and high S-phase (Fig. 3B). Meta-cluster 13 may be associated with disease relapse and suggests that patients with elevated meta-cluster 13 may benefit from anti-proliferative therapy. Further, samples associated with high mSMART also had a high proportion of meta-cluster 9, characterized by elevated cell surface transferrin receptor CD71, and pERK suggesting targeting CD71 may be a promising therapeutic strategy in these patients. Samples with high S-phase had an increased abundance of meta-cluster 2, characterized by elevated cell surface CD81 expression, another potential therapeutic target. Meta-cluster 8, characterized by the highest relative expression of CD38, was significantly reduced in RRMM samples and in those with *TP53* deletion and high S-phase, possibly driven by daratumumab treatment.

Since CD38 was significantly lower in daratumumab-treated vs. daratumumab-naïve samples (Fig. 1C), we investigated whether differences in the phenotypic profiles of CD38-driven meta-clusters were skewed due to daratumumab. We repeated clustering analysis and meta-cluster classification with the exclusion of CD38. As before, we determined significant markers with relative increases or decreases in expression in each cluster and compared the meta-clusters that had been analyzed with CD38 inclusion to those with CD38 exclusion (Supplementary Fig. 4A). In comparison to the previous analysis, in which 13 different phenotypic meta-clusters were identified, reanalysis without CD38 revealed 12 phenotypic meta-clusters. The expression characteristics of these 12 phenotypic meta-clusters were remarkably similar, suggesting that within these 12 meta-clusters, the influence of CD38 on meta-cluster assignment was minimal. Meta-cluster 8, characterized in the original analysis as having the highest CD38 expression (Supplementary Table 5), was the only meta-cluster lost in the reanalysis suggesting that CD38 played a significant role in the classification of meta-cluster 8. Approximately 11% of the cells belonging to meta-cluster 8 in the previous analysis were redistributed to other meta-clusters (Supplementary Fig. 4A). Similar to the original analysis, meta-cluster 13, with increased Ki-67 and CD117, remained significantly more prevalent in samples with 1q gain and elevated mSMART. Samples associated with a poor depth of response retained their reduced abundance of meta-cluster 1 in the reanalysis (Supplementary Fig. 4B).

We next compared whether the presence or absence of each of the 13 phenotypic meta-clusters within 6 months of MM diagnosis was predictive of overall survival (OS) in our patient cohort. The only phenotypic meta-cluster associated with a significant difference in OS was meta-cluster 1 (Fig. 3C). Among patients with an absence of phenotypic meta-cluster 1, OS was decreased compared to patients with the presence of phenotypic meta-cluster 1 (2.2 years vs. 9.4 years, *P* = 0.017) characterized by increased CD45 and reduced BCL-2 expression. Consistent with these findings, the presence of phenotypic meta-cluster 1 was associated with improved OS (RR 0.21 (0.05–0.84), *P* = 0.027) (Supplementary Table 6). A similar distribution of mSMART risk status was observed in association with the presence (52.9%) or absence (57.1%) of phenotypic meta-cluster 1 (*P* = 0.67) (Fig. 3D). In a multivariate model including the presence of meta-cluster 1 and standard risk mSMART status, meta-cluster 1 retained its trend toward improved OS (RR 0.28 (0.07–1.08), *P* = 0.063) (Supplementary Table 6).

**Fig. 4:**
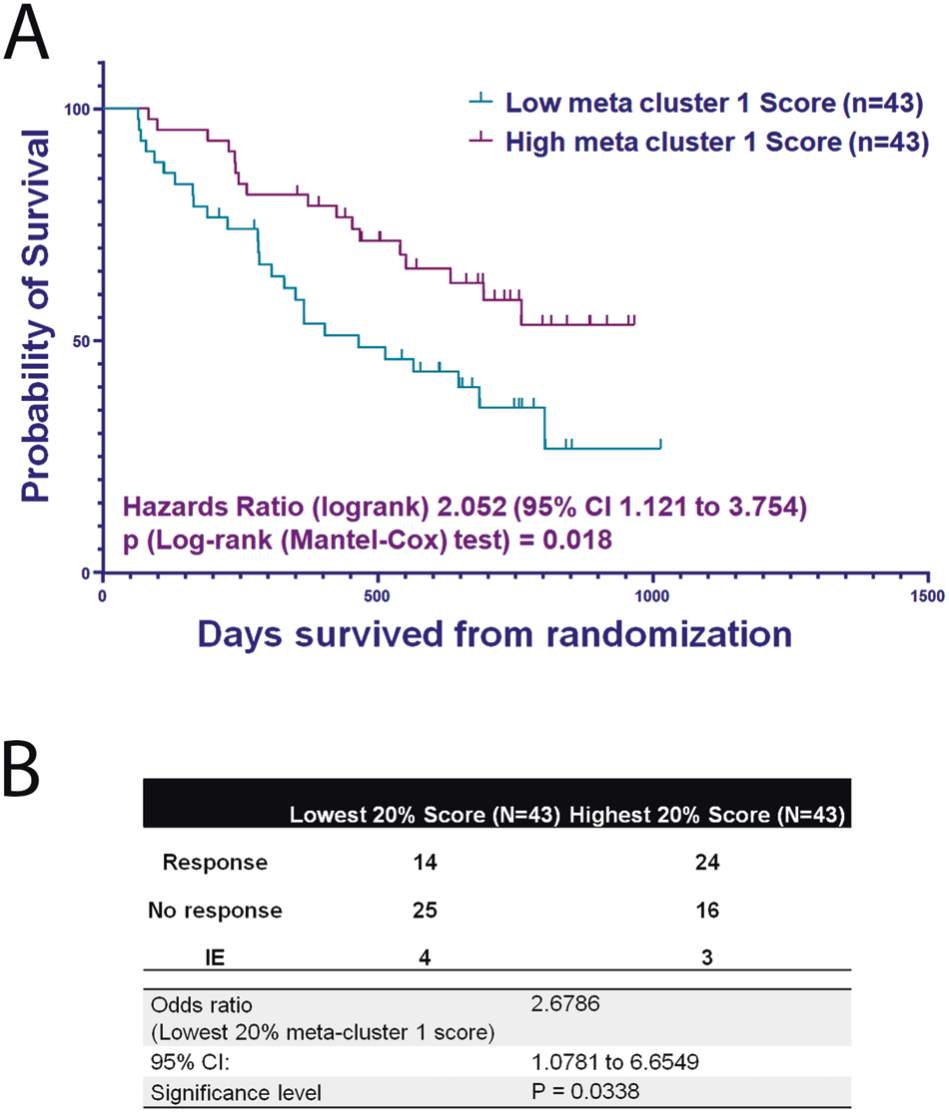
Impact of Meta-cluster 1 score on overall survival in the APEX trial. **A** A comparison of OS (days) in patients with low meta-cluster 1 (green line) and in patients with high meta-cluster 1 (purple line). **B** Top and bottom 20% meta-cluster 1 scores compared to responder status.

We sought to validate the association of meta-cluster 1 with a survival benefit in a larger patient cohort. Since an external CyTOF dataset is not available, we utilized gene expression and clinical outcome data from the APEX phase-3 MM clinical trial. Median gene intensity scores were calculated for the genes that defined the meta-cluster 1 protein signature: high *PTPRC* (CD45) and low *BCL-2* as well as high *NFKBIA* (IkBα), *MYC* and and *IKZF3*. A combined meta-cluster 1 activity score was computed for each sample as the summation of the ascending ranks of expression of median gene intensities. To determine whether the meta-cluster 1 activity score was associated with clinical outcomes, we performed survival analysis between patients with the highest vs. the lowest meta-cluster 1 score. Patients with a low meta-cluster 1 score were associated with poorer OS in comparison to patients with a high meta-cluster 1 score (Log-rank hazards ratio = 2.052 (95% CI 1.121–3.754; *p* = 0.018) (Fig. 4). Further, the odds of no response (NR) vs. response (R) between the patients with the lowest meta-cluster 1 score vs. the highest meta-cluster 1 score was 2.6786 (PGx responder status vs. meta-cluster 1 score; Odds ratio (OR) = 2.6786; *P* = 0.0338; 95% CI 1.0781 to 6.6549), indicating a low meta-cluster 1 score was associated with > 2.5-fold higher cancer progression in MM patients compared to a high meta-cluster 1 score.

## Discussion

Here we used single-cell CyTOF to characterize MM tumor heterogeneity among 49 patient samples. Our goal was to detect shared MM subpopulations associated with treatment response and disease outcome across all patient samples. We employed differential expression analysis to group similar clusters within each patient sample so that we could compare phenotypic meta-clusters among all patients. Using a random forest model, we identified cells with similar marker expression patterns, allowing us to identify 13 unique phenotypic meta-clusters. To the best of our knowledge, this is the first study to evaluate subclonal heterogeneity, characterized by the analysis of ~40 phenotypic cell surface and intracellular markers using primary MM samples. Despite significant heterogeneity, we found common patterns of subclonal protein profiles that were shared across patients and are correlated with clinical behavior. Evaluation of tumor proteomic profiles may provide an opportunity for the identification of novel protein-based biomarkers associated with disease state and therapeutic response and may even open the possibility for innovative protein-based therapeutic interventions.

Of the 13 phenotypic meta-clusters identified, meta-cluster 1 was the only meta-cluster that had a significant impact on response to therapy and overall disease outcomes. This impact was validated using the APEX 3 clinical trial. Meta-cluster 1 was primarily characterized by a subpopulation of MM cells with increased CD45 and reduced BCL-2 expression. Reduced abundance of meta-cluster 1 was associated with poor treatment response and survival. This observation is consistent with previous studies demonstrating that patients with CD45 negative MM had poorer OS compared to patients with CD45 positive MM, suggesting that the CD45 negative MM phenotype may be a marker of progressive disease [48, 49]. CD45 low or negative MM cells have also been identified to have a higher engraftment ability in mice compared to CD45 positive MM cells [50]. Of interest, the CD45 positive MM cells have been previously associated with low expression of the pro-survival protein BCL-2 along with an increased susceptibility to apoptosis [51], suggesting that phenotypic meta-cluster 1 may be more sensitive to treatment-related cell death. Our work also revealed increased BCL-2 expression in RRMM, a finding that has previously not been reported and may suggest an increased sensitivity to venetoclax in this patient cohort. Further, since we show that BCL-2 expression correlates with t(11;14), the increased BCL-2 expression in RRMM was not a reflection of a higher frequency of t(11;14) in the RRMM group (NDMM: 35% t(11;14) vs. RRMM: 28% t(11;14)).

Consistent with previous studies [41, 52], we found that the monoclonal CD38 antibody daratumumab interfered with the CD38 antibody used in our CyTOF studies, precluding the reliable use of CD38 for initial gating approaches and in the downstream analysis of patients treated with daratumumab within 6 months prior to sample collection. Remarkably, the exclusion of CD38 in the reanalysis of our data appeared to primarily impact the identification of phenotypic meta-cluster 8, characterized by the highest CD38 expression, and the remaining 12 clusters maintained their phenotype identity independent of CD38 expression. Future studies using alternative CD38 epitopes that do not compete with daratumumab should be conducted. Although the overall detection of surface CD38 was lower in samples obtained from patients treated with daratumumab than in untreated patients (Fig. 1C), two patients represented by the highest two data points in the treatment cohort did not have reduced CD38 detection 2 and 4-months post treatment. Whether this was a result of altered CD38 expression [40, 41] or a lack of antibody interference by daratumumab remains unknown [41].

This study is limited by its retrospective nature owing to the use of cryopreserved samples for CyTOF analysis and heterogeneity in treatment regimens in the RRMM group (Supplementary Table 4). Despite these limitations, we showed that MM samples display a significant degree of complexity in protein expression, but that subclonal structure is shared among patients. We identified 13 unique phenotypic meta-clusters across the 49 MM samples. Several of the meta-clusters were associated with unique clinical features and disease subtypes. The marker proteins of each phenotypic meta-cluster may serve as therapeutic targets or biomarkers which could be evaluated in future studies.

## Supplementary information

Supplementary Tables

Supplementary Table 5

Supplementary Methods

Supplementary Figures

## Data Availability

Requests regarding data availability not already shared in the supplementary material should be made to the corresponding author.
