# Supplementary material for: Mass Cytometry reveals unique phenotypic patterns associated with subclonal diversity and outcomes in multiple myeloma
Source: Blood Cancer J. 2023 May 22;13(1):84. doi: 10.1038/s41408-023-00851-5 (PMC10203138; doi:10.1038/s41408-023-00851-5)
Supplement: Supplementary file 1 — Supplementary Tables [file 41408_2023_851_MOESM1_ESM.docx]

| **Cell surface targets** | **Metal Tag** | **Source** | **Catalog No.** |
| --- | --- | --- | --- |
| CD45 | 89Y | Fluidigm | 3089003B |
| CD38 | 114Nd | Fluidigm | 3144014B |
| CD138 | 168Er | Fluidigm | 3168009B |
| CD56 | 149Sm | Fluidigm | 3149021B |
| CD81 | 145Nd | Fluidigm | 3145007B |
| CD20 | 147Sm | Fluidigm | 3147001B |
| CD34 | 148Nd | Fluidigm | 3148001B |
| CD274 (PD-L1) | 159Tb | Fluidigm | 3159029B |
| CD27 | 167Er | Fluidigm | 3167006B |
| CD49d | 174Yb | Fluidigm | 3174018B |
| CD117* | 173Yb | BioLegend | 313223 |
| CD28* | 154Sm | BioLegend | 302937 |
| CD147* | 161Dy | BioLegend | 306206 |
| CD71* | 170Er | BioLegend | 334102 |
| CD3 | 141Pr | Fluidigm | 3141019B |
| CD19 | 169Tm | Fluidigm | 3169011B |
| CD16 | 209Bi | Fluidigm | 3209002B |
| **Intracellular targets** | **Metal Tag** | **Source** | **Catalog No.** |
| IkBα | 164Dy | Fluidigm | 3164004A |
| SOX2 | 150Nd | Fluidigm | 3150019B |
| MCL1 | 163Dy | Fluidigm | 3163006A |
| IKZF3 (Aiolos) | 162Dy | Fluidigm | 3162032B |
| IRF4 (MUM-1) | 155Gd | Fluidigm | 3155014B |
| IKZF1 (Ikaros) | 143Nd | Fluidigm | 3143024B |
| Myc | 176Yb | Fluidigm | 3176012B |
| BCL-2* | 153Eu | BioLegend | 658702 |
| CRBN* (Cereblon) | 146Nd | Sigma | SAB1407456 |
| Ki-67 | 172Yb | Fluidigm | 3172024B |
| pS6 [S235/S236] | 175Lu | Fluidigm | 3175009A |
| pAkt [S473] | 152Sm | Fluidigm | 3152005A |
| pP38 [T180/Y182] | 156Gd | Fluidigm | 3156002A |
| pRb [S807/811] | 166Er | Fluidigm | 3166011A |
| pCREB [S133] | 165Ho | Fluidigm | 3165009A |
| pERK 1/2 [T202/Y204] | 171Yb | Fluidigm | 3171010A |
| pSTAT3 [Y705] | 158Gd | Fluidigm | 3158005A |
| Caspase 3/Cleaved | 142Nd | Fluidigm | 3142004A |
| Ig kappa/light chain | 160Gd | Fluidigm | 3160005B |
| Ig lambda/light chain | 151Eu | Fluidigm | 3151004B |

**Supplementary Table 1**: Antibodies used for CyTOF processing.

*Antibodies conjugated using the X8 polymer MaxPAR kit (Fluidigm) as per manufacturer’s instructions.

**Supplementary Table 2**: Reagents and their sources

| **Reagents** | - **Source** | - **Catalog number** |
| --- | --- | --- |
| - Benzonase | - Sigma Aldrich | - E1014 |
| - Cell-ID Cisplatin | - Fluidigm | - 201064 |
| - Maxpar Fix I Buffer | - Fluidigm | - 201065 |
| - Veri-Cells PBMC | - BioLegend | - 425003 |
| - Maxpar Cell Staining Buffer (CSB) | - Fluidigm | - 201068 |
| - Formaldehyde solution | - Thermo Scientific | - 28906 |
| - Cell-ID Intercalator-Ir | - Fluidigm | - 201192A |
| - Maxpar Fix and Perm Buffer | - Fluidigm | - 201067 |
| - Maxpar Cell Acquisition Solution | - Fluidigm | - 201237 |
| - Cell-ID 20-Plex Pd Barcoding Kit | - Fluidigm | - 201060 |

CyTOF specific reagents utilized during sample processing. Maxpar reagents, including water, Cell-ID Cisplatin, Fix and Perm Buffer, Cell Staining Buffer (CSB), Cell-ID Intercalator-Ir, Cell-ID 20-Plex Pd Barcoding Kit, Cell Acquisition Solution, and EQ Four Element Calibration Beads, were purchased from Fluidigm.

**Supplementary Table 3**: Cell surface and intracellular markers included in this study and their association with MM disease.

| **Cell surface targets** | **Association of bulk protein or gene expression with poor MM disease outcome, drug resistance or relapse** | | | |
| --- | --- | --- | --- | --- |
| CD45 | (1-4) |  |  |  |
| CD38 | (5) |  | | |
| CD138 | (6) |  | | |
| CD56 | (4, 7) |  |  | |
| CD81 | (8) |  | | |
| CD20 | (4) |  | | |
| CD34 | (9) |  | | |
| CD274 (PD-L1) | (10) |  | | |
| CD27 | (11-13) |  | | |
| CD49d | (14) |  | | |
| CD117* | (4) | with CD28 |  | |
| CD28* | (4) |  | | |
| CD147* | (15) |  | | |
| CD71* | (16) |  | | |
| IkBα | (17, 18) |  | | |
| SOX2 | (19) |  | | |
| MCL1 | (20) |  | | |
| IKZF3 (Aiolos) | (21, 22) |  |  | |
| IRF4 | (22, 23) |  |  | |
| IKZF1 (Ikaros) | (21, 22) |  |  | |
| Myc | (24) |  | | |
| BCL-2* | (25) | Venetoclax response |  | |
| CRBN* (Cereblon) | (22) |  | | |
| Ki-67 | (26) |  | | |
| pS6 [S235/S236] | (27) |  | | |
| pAkt [S473] | (28) |  | | |
| pP38 [T180/Y182] | (29-31) |  | | |
| pRb [S807/811] | (32) |  | | |
| pCREB [S133] | (33) |  | | |
| pERK 1/2 [T202/Y204] | (28, 34) |  |  | |
| pSTAT3 [Y705] | (28, 35) |  |  | |

Association of bulk protein or gene expression of each marker evaluated in this study (as detected by flow cytometry or immunohistochemistry) to multiple myeloma (MM) disease outcome, drug resistance or disease relapse. Red is associated with elevated protein expression, blue is associated with reduced protein expression, grey is associated with no change in protein expression and brown is associated with uncertain protein expression in association with either poor MM disease outcome, drug resistance or relapse. Multiple colors indicate variable significance of marker to outcome. Study reference indicated.

**Supplementary Table 4:** Patient treatment

| Patient | Treatment |
| --- | --- |
| 1 | None-NDMM |
| 2 | None-NDMM |
| 3 | None-NDMM |
| 4 | None-NDMM |
| 5 | None-NDMM |
| 6 | None-NDMM |
| 7 | None-NDMM |
| 8 | None-NDMM |
| 9 | None-NDMM |
| 10 | None-NDMM |
| 11 | None-NDMM |
| 12 | None-NDMM |
| 13 | None-NDMM |
| 14 | None-NDMM |
| 15 | None-NDMM |
| 16 | None-NDMM |
| 17 | None-NDMM |
| 19 | None-NDMM |
| 19 | None-NDMM |
| 20 | None-NDMM |
| 21 | KRD |
| 22 | VRD+Elo+Ixa+KD+*Dara***+Pom |
| 23 | RD+ASCT+*Dara* (after collection) |
| 24 | melphalan+pred+thal+selinexor+*Dara*** |
| 25 | CyBorD +VRD+ASCT+VRD+*Dara*** |
| 26 | melphalan+pred+thal+selinexor+ *Dara* (after collection) |
| 27 | CyBorD +ASCT+*Dara*** |
| 28 | CyBorD +ASCT+R+*Dara* (after collection) |
| 29 | CyBorD +VRD+*Dara*** |
| 30 | CyBorD +ASCT+*Dara* (after collection) |
| 31 | RD+ASCT+ *Dara* (after collection) |
| 32 | melphalan+pred+thal+selinexor+*Dara** |
| 33 | VR |
| 34 | RD for SMM, VD+*Dara*** |
| 35 | RD+ASCT+*Dara*** |
| 36 | VD+Ben+CyBorD+ASCT+*Dara* (after collection) |
| 37 | RD |
| 38 | RD+ASCT+*Dara*** |
| 39 | VRD+ASCT+KRD |
| 40 | CyBorD +ASCT+R+*Dara*** |
| 41 | VD+VTD+VRD+CTX+pred+ASCT+*Dara* (after collection) |
| 42 | VD+Ben+CyBorD+ASCT+*Dara*** |
| 43 | VRD+R+*Dara** |
| 44 | CRD+*Dara** |
| 45 | VRD+KPD+*Dara***+Pom+Dex |
| 46 | VRD+ASCT+R+Ixa+Pom+D+*Dara* (after collection) |
| 47 | RD+ASCT+R+Ixa+D+pembro+Pom+*Dara***+V+D |
| 48 | VD+VRD+ASCT+R+*Dara*** |
| 49 | KRD+adriamycin-D+cytoxan+K+Dara**+Pom+Nelfinavir |

Treatment at any time of the patients in this study: thal (thalidomide); CyBorD (cyclophosphamide, bortezomib (Velcade), dexamethasone); VTD (Velcade, thalidomide, dexamethasone); VRD (Velcade, Revlimid (lenalidomide), dexamethasone); CRD (cyclophosphamide, Revlimid, dexamethasone); KRD (Carfilzomib, Revlimid, dexamethasone); Elo (elotuzumab); Pom (pomalidomide); Ixa (ixazomib); ben (bendamustine); pembro (pembrolizumab); pom (pomalidomide); pred (prednisone); ASCT (autologous stem cell transplant); dara (daratumumab: (after collection), * (>6 months prior to sample collection), ** (< 6 months prior to sample collection).

**Supplementary Table 6:** Risk ratios of each of the 13 phenotypic meta-clusters in association with overall survival (OS).

| **Meta-cluster**  **(yes vs. no)** | **Risk Ratio** | **P-value** | **Lower 95%** | **Upper 95%** |  |  |  |  |
| --- | --- | --- | --- | --- | --- | --- | --- | --- |
| 1 | 0.21 | 0.027 | 0.05 | 0.84 |  |  |  |  |
| 2 | 0.89 | 0.849 | 0.27 | 2.93 |  |  |  |  |
| 3 | 1.36 | 0.611 | 0.41 | 4.47 |  |  |  |  |
| 4 | 2.74 | 0.203 | 0.57 | 12.96 |  |  |  |  |
| 5 | 0 | 0.998 | 0 | N/A |  |  |  |  |
| 6 | 0.51 | 0.310 | 0.14 | 1.84 |  |  |  |  |
| 7 | 1.28 | 0.748 | 0.27 | 6.09 |  |  |  |  |
| 8 | 1.87 | 0.341 | 0.51 | 6.84 |  |  |  |  |
| 9 | 0.88 | 0.854 | 0.22 | 3.43 |  |  |  |  |
| 10 | 0.62 | 0.459 | 0.18 | 2.16 |  |  |  |  |
| 11 | 1.08 | 0.895 | 0.31 | 3.88 |  |  |  |  |
| 12 | 1.53 | 0.542 | 0.38 | 6.08 |  |  |  |  |
| 13 | 1.55 | 0.498 | 0.43 | 5.51 |  |  |  |  |
|  |  |  |  |  |  |  |  |  |
|  | **Univariate model** | | | | **Multivariate model** | | | |
| **Independent variables** | **Risk Ratio** | **P-value** | **Lower 95%** | **Upper 95%** | **Risk Ratio** | **P-value** | **Lower 95%** | **Upper 95%** |
| Meta-cluster 1  (yes vs. no) | 0.21 | 0.027 | 0.05 | 0.84 | 0.28 | 0.063 | 0.07 | 1.08 |
| mSMART  (standard vs. high) | 0.21 | 0.045 | 0.04 | 0.96 | 0.25 | 0.079 | 0.05 | 1.17 |

Top) Comparison of the presence or absence of the 13 phenotypic meta-clusters on OS on univariate analysis. Bottom) Comparison of the presence or absence of meta-cluster 1 or mSMART status on OS on univariate analysis and multivariate analysis. P-value determined using Wald test. Risk ratios are considered significant if p<0.05.

**References**

1. Moreau P, Robillard N, Avet-Loiseau H, Pineau D, Morineau N, Milpied N, et al. Patients with CD45 negative multiple myeloma receiving high-dose therapy have a shorter survival than those with CD45 positive multiple myeloma. Haematologica. 2004;89(5):547-51.

2. Kumar S, Rajkumar SV, Kimlinger T, Greipp PR, Witzig TE. CD45 expression by bone marrow plasma cells in multiple myeloma: clinical and biological correlations. Leukemia. 2005;19(8):1466-70.

3. Gonsalves WI, Timm MM, Rajkumar SV, Morice WG, Dispenzieri A, Buadi FK, et al. The prognostic significance of CD45 expression by clonal bone marrow plasma cells in patients with newly diagnosed multiple myeloma. Leuk Res. 2016;44:32-9.

4. Mateo G, Montalban MA, Vidriales MB, Lahuerta JJ, Mateos MV, Gutierrez N, et al. Prognostic value of immunophenotyping in multiple myeloma: a study by the PETHEMA/GEM cooperative study groups on patients uniformly treated with high-dose therapy. J Clin Oncol. 2008;26(16):2737-44.

5. Seckinger A, Hillengass J, Emde M, Beck S, Kimmich C, Dittrich T, et al. CD38 as Immunotherapeutic Target in Light Chain Amyloidosis and Multiple Myeloma-Association With Molecular Entities, Risk, Survival, and Mechanisms of Upfront Resistance. Front Immunol. 2018;9:1676.

6. Kawano Y, Fujiwara S, Wada N, Izaki M, Yuki H, Okuno Y, et al. Multiple myeloma cells expressing low levels of CD138 have an immature phenotype and reduced sensitivity to lenalidomide. Int J Oncol. 2012;41(3):876-84.

7. Sahara N, Takeshita A, Shigeno K, Fujisawa S, Takeshita K, Naito K, et al. Clinicopathological and prognostic characteristics of CD56-negative multiple myeloma. Br J Haematol. 2002;117(4):882-5.

8. Paiva B, Gutierrez NC, Chen X, Vidriales MB, Montalban MA, Rosinol L, et al. Clinical significance of CD81 expression by clonal plasma cells in high-risk smoldering and symptomatic multiple myeloma patients. Leukemia. 2012;26(8):1862-9.

9. Kuranda K, Berthon C, Dupont C, Wolowiec D, Leleu X, Polakowska R, et al. A subpopulation of malignant CD34+CD138+B7-H1+ plasma cells is present in multiple myeloma patients. Exp Hematol. 2010;38(2):124-31.

10. Lee BH, Park Y, Kim JH, Kang KW, Lee SJ, Kim SJ, et al. PD-L1 expression in bone marrow plasma cells as a biomarker to predict multiple myeloma prognosis: developing a nomogram-based prognostic model. Sci Rep. 2020;10(1):12641.

11. Guikema JE, Hovenga S, Vellenga E, Conradie JJ, Abdulahad WH, Bekkema R, et al. CD27 is heterogeneously expressed in multiple myeloma: low CD27 expression in patients with high-risk disease. Br J Haematol. 2003;121(1):36-43.

12. Alaterre E, Raimbault S, Goldschmidt H, Bouhya S, Requirand G, Robert N, et al. CD24, CD27, CD36 and CD302 gene expression for outcome prediction in patients with multiple myeloma. Oncotarget. 2017;8(58):98931-44.

13. Chu B, Bao L, Wang Y, Lu M, Shi L, Gao S, et al. CD27 antigen negative expression indicates poor prognosis in newly diagnosed multiple myeloma. Clin Immunol. 2020;213:108363.

14. Paiva B, Corchete LA, Vidriales MB, Puig N, Maiso P, Rodriguez I, et al. Phenotypic and genomic analysis of multiple myeloma minimal residual disease tumor cells: a new model to understand chemoresistance. Blood. 2016;127(15):1896-906.

15. Lacina P, Butrym A, Turlej E, Stachowicz-Suhs M, Wietrzyk J, Mazur G, et al. BSG (CD147) Serum Level and Genetic Variants Are Associated with Overall Survival in Acute Myeloid Leukaemia. J Clin Med. 2022;11(2).

16. Deng QW, Zhang J, Li T, He WM, Fang L, Lee HC, et al. The transferrin receptor CD71 regulates type II CD38, revealing tight topological compartmentalization of intracellular cyclic ADP-ribose production. J Biol Chem. 2019;294(42):15293-303.

17. Demchenko YN, Glebov OK, Zingone A, Keats JJ, Bergsagel PL, Kuehl WM. Classical and/or alternative NF-kappaB pathway activation in multiple myeloma. Blood. 2010;115(17):3541-52.

18. Annunziata CM, Davis RE, Demchenko Y, Bellamy W, Gabrea A, Zhan F, et al. Frequent engagement of the classical and alternative NF-kappaB pathways by diverse genetic abnormalities in multiple myeloma. Cancer Cell. 2007;12(2):115-30.

19. Spisek R, Kukreja A, Chen LC, Matthews P, Mazumder A, Vesole D, et al. Frequent and specific immunity to the embryonal stem cell-associated antigen SOX2 in patients with monoclonal gammopathy. J Exp Med. 2007;204(4):831-40.

20. Wuilleme-Toumi S, Robillard N, Gomez P, Moreau P, Le Gouill S, Avet-Loiseau H, et al. Mcl-1 is overexpressed in multiple myeloma and associated with relapse and shorter survival. Leukemia. 2005;19(7):1248-52.

21. Pourabdollah M, Bahmanyar M, Atenafu EG, Reece D, Hou J, Chang H. High IKZF1/3 protein expression is a favorable prognostic factor for survival of relapsed/refractory multiple myeloma patients treated with lenalidomide. J Hematol Oncol. 2016;9(1):123.

22. Misiewicz-Krzeminska I, de Ramon C, Corchete LA, Krzeminski P, Rojas EA, Isidro I, et al. Quantitative expression of Ikaros, IRF4, and PSMD10 proteins predicts survival in VRD-treated patients with multiple myeloma. Blood Adv. 2020;4(23):6023-33.

23. Bai H, Wu S, Wang R, Xu J, Chen L. Bone marrow IRF4 level in multiple myeloma: an indicator of peripheral blood Th17 and disease. Oncotarget. 2017;8(49):85392-400.

24. Chng WJ, Huang GF, Chung TH, Ng SB, Gonzalez-Paz N, Troska-Price T, et al. Clinical and biological implications of MYC activation: a common difference between MGUS and newly diagnosed multiple myeloma. Leukemia. 2011;25(6):1026-35.

25. Bodet L, Menoret E, Descamps G, Pellat-Deceunynck C, Bataille R, Le Gouill S, et al. BH3-only protein Bik is involved in both apoptosis induction and sensitivity to oxidative stress in multiple myeloma. Br J Cancer. 2010;103(12):1808-14.

26. Alexandrakis MG, Passam FH, Kyriakou DS, Dambaki K, Niniraki M, Stathopoulos E. Ki-67 proliferation index: correlation with prognostic parameters and outcome in multiple myeloma. Am J Clin Oncol. 2004;27(1):8-13.

27. Chen X, Diaz-Rodriguez E, Ocio EM, Paiva B, Mortensen DS, Lopez-Girona A, et al. Genetic and pharmacologic evidence that mTOR targeting outweighs mTORC1 inhibition as an antimyeloma strategy. Mol Cancer Ther. 2014;13(2):504-16.

28. Brown R, Yang S, Weatherburn C, Gibson J, Ho PJ, Suen H, et al. Phospho-flow detection of constitutive and cytokine-induced pSTAT3/5, pAKT and pERK expression highlights novel prognostic biomarkers for patients with multiple myeloma. Leukemia. 2015;29(2):483-90.

29. Hideshima T, Akiyama M, Hayashi T, Richardson P, Schlossman R, Chauhan D, et al. Targeting p38 MAPK inhibits multiple myeloma cell growth in the bone marrow milieu. Blood. 2003;101(2):703-5.

30. He J, Liu Z, Zheng Y, Qian J, Li H, Lu Y, et al. p38 MAPK in myeloma cells regulates osteoclast and osteoblast activity and induces bone destruction. Cancer Res. 2012;72(24):6393-402.

31. Yang J, He J, Wang J, Cao Y, Ling J, Qian J, et al. Constitutive activation of p38 MAPK in tumor cells contributes to osteolytic bone lesions in multiple myeloma. Leukemia. 2012;26(9):2114-23.

32. Ely S, Di Liberto M, Niesvizky R, Baughn LB, Cho HJ, Hatada EN, et al. Mutually exclusive cyclin-dependent kinase 4/cyclin D1 and cyclin-dependent kinase 6/cyclin D2 pairing inactivates retinoblastoma protein and promotes cell cycle dysregulation in multiple myeloma. Cancer Res. 2005;65(24):11345-53.

33. Liu Z, Li T, Jiang K, Huang Q, Chen Y, Qian F. Induction of chemoresistance by all-trans retinoic acid via a noncanonical signaling in multiple myeloma cells. PLoS One. 2014;9(1):e85571.

34. Fan L, Hong J, Huang H, Fu D, Wu S, Wang Q, et al. High Expression of Phosphorylated Extracellular Signal-Regulated Kinase (ERK1/2) is Associated with Poor Prognosis in Newly Diagnosed Patients with Multiple Myeloma. Med Sci Monit. 2017;23:2636-43.

35. Teoh PJ, Chung TH, Chng PYZ, Toh SHM, Chng WJ. IL6R-STAT3-ADAR1 (P150) interplay promotes oncogenicity in multiple myeloma with 1q21 amplification. Haematologica. 2020;105(5):1391-404.
