# Supplementary material for: Mass Cytometry reveals unique phenotypic patterns associated with subclonal diversity and outcomes in multiple myeloma
Source: Blood Cancer J. 2023 May 22;13(1):84. doi: 10.1038/s41408-023-00851-5 (PMC10203138; doi:10.1038/s41408-023-00851-5)
Supplement: Supplementary file 3 — Supplementary Methods [file 41408_2023_851_MOESM3_ESM.docx]

**Supplemental Materials and Methods**

**Fluorescence in Situ Hybridization (FISH)**

FISH analysis of immunoglobulin (cIg)-stained positive PCs studies were performed using the following probes to detect primary and secondary MM abnormalities: monosomy 13 or 13q deletion (Abbott Molecular, Des Plaines, IL), monosomy 17 or *TP53* deletion (Abbott Molecular), trisomy 3, 7, 9 or 15 (Abbott Molecular), 1q22 gain (in house, custom developed), *MYC* rearrangement (Abbott Molecular), IgH rearrangement (in house, custom developed), t(11;14)(q13;q32) *CCND1::IgH* (Abbott Molecular), t(4;14)(p16.3;q32) *FGFR3::IgH* (Abbott Molecular), t(6;14)(p21;q32) *CCND3::IgH* (Abbott Molecular), t(14;16)(q32;q23) *IgH::MAF* (Abbott Molecular), and t(14;20)(q32;q12) *IgH::MAFB* (Abbott Molecular)*.* The genomic location of each probe is described in [1].

**Treatment Response Asssesment**

Complete response (CR) was defined as the absence of monoclonal protein or M protein in the serum and urine by immunofixation, with no evidence of soft tissue plasmacytoma and bone marrow demonstrating less than 5% clonal PCs. Patients who did not undergo bone marrow biopsy at the time of evaluation were deemed to have a complete hematological response. In those who lacked M proteins in the serum and urine and were monitored by FLC levels, CR was defined by normalization of FLC levels.  A very good partial response (VGPR) was defined as a 90% reduction in serum M protein, with urine M protein <100 mg/24 hours. In those who lacked M proteins in the serum and urine and were monitored by FLC levels, VGPR was defined as a 90% reduction in the difference between involved and uninvolved light chains. The partial response was defined as more than a 50% reduction in serum M protein or a ≥ 50% decrease in the difference between involved and uninvolved FLC. Progressive disease was defined as a 24% increase from the lowest value in serum M protein or difference in FLC.

**Sample preparation for CyTOF**

For CyTOF cell staining, 1-3 million cells were washed using RPMI1640 with 20% FBS and incubated with Cell-ID. Cell-ID cisplatin (1:10,000 dilutions in RPMI1640 media without FBS) was added at room temperature (RT). Cells were resuspended in 1x Maxpar Fix and Perm Buffer and incubated for 10 min at RT. Along with staining of the patient samples, two internal reference control samples of unstimulated and cytokine-activated cells to signal to the MAPK/ERK pathway (treated with phorbol 12-myristate 13-acetate (PMA) and ionomycin for 15 min) were also used. These Veri-Cell PBMC lyophilized reference controls (BioLegend) were washed using Maxpar CSB. An antibody cocktail of the surface panel was prepared, added to each tube containing the cell suspension in 100 µL CSB and incubated for 30 min at RT. Cells were washed with CSB and stored overnight at -80°C after methanol fixation. An antibody cocktail for the intracellular panel was prepared. Cells were washed with CSB and incubated with 20 µL of an intracellular antibody cocktail for 30 min at RT. Cells were fixed with a fresh 1.6% formaldehyde solution (Thermo Fisher Scientific), washed with CSB and resuspended in 1 ml of Cell-ID Intercalator-Lr solution (1:4000 dilution in Maxpar Fix and Perm Buffer). Samples were barcoded using the Cell-ID 20-Plex Pd Barcoding Kit and combined into a single multiplexed tube along with the Maxpar cell acquisition solution and EQ four-element calibration beads followed by filtration through a 35 µm blue cap Falcon tube (Thermo Fischer Scientific). Samples were acquired on the Helios CyTOF system using an attached autosampler at a rate of 200-400 events per second in the Mayo Clinic Immune Monitoring Core. Data were collected as flow cytometry standard (FCS) files using the CyTOF software (Fluidigm, Version 6.7.1014). After acquisition, the intra-file signal drift was debarcoded and normalized to the acquired calibration bead signal.

**Processing of CyTOF Flow Cytometry Standard (FCS) files**

Normalized FCS files from the Mayo Clinic Immune Monitoring Core were analyzed by the Maxpar Pathsetter software (version 2.0; Fluidigm) for cleanup to select live cell events. Live cell events were classified according to Fluidigm’s cleanup model as having 1) negative bead signal; 2) largest population of Bayesian variables offset, width, center, residual, and event length; 3) negative cisplatin signal; and 4) largest population of DNA1 and DNA2 signals (^191^lr and ^193^lr). Events that passed the filtering criteria were exported to a new FCS file for downstream processing.

**Cell Modeling**

Cleaned FCS files containing live cell events were analyzed by the Maxpar Pathsetter software for classification as either PC kappa^+^, PC lambda^+^, or unclassified. Markers were classified into three categories: selection, staging, and functional. Selection markers were the only markers used to define the cell type. Internal peak-finding algorithms in Maxpar Pathsetter were used to classify events as positive or negative for a marker. PC kappa^+^ events were described as CD3^-^, CD16^-^, CDH19^-^, CD138^+^, IRF4 (MUM-1)^+^ and kappa^+^. PC lambda^+^ events were described as CD3^-^, CD16^-^, CD19^-^, CD138^+^, IRF4^+^ and lambda^+^ (**Supplementary Figure 1D**). The average surface expression of CD138 was low because cryopreservation and sample processing are known to be associated with reduced CD138 surface expression [2]. CD38 was not included in the classification model because of known exposure to daratumumab in some patients. The PC identification approach was reviewed by a haematopathologist (M.A.L). The events assigned to PC kappa^+^ and PC lambda^+^ were exported to new FCS files. The criteria for inclusion in further analysis required having at least 500 live light-chain-restricted PCs (median 3710, range 687–90807) (**Supplementary Figure 1E**). The distribution of marker abundance between samples run in different batches was normalized by performing rank-based quantile normalization using the statistical program R v3.6.2.

**Data Processing**

For bulk sample-level processing, the average abundance of each marker within a sample was determined by averaging the marker abundance for all cells. The analysis was performed using in-house Perl and R scripts.

For subpopulation processing, unsupervised clustering was performed using the Rphenograph (0.99.1) package in R v3.6.2. The default k-value of 30 was used as the clustering threshold. The resulting number of clusters for each sample ranged from 11 to 29**.**

Next, the expression of differentially abundant markers between clusters in a sample was determined using the MAST (1.18) package in the R v3.6.2. Each cluster was compared to all other cells, and not within the cluster. MAST reported Z-scores show the degree and directionality of the change in expression, p-value, and false discovery rate (FDR). The proportion of cells with changes in a marker, either increased (positive Z-score) or decreased (negative Z-score), was calculated as the summation of the number of cells assigned to each cluster with the corresponding change.

For cross sample correlations, individual patient PC clusters were divided into meta-clusters using the MAST Z-scores for all markers as inputs for unsupervised phenograph clustering to identify cluster-matched equivalents among all patients. The average correlation of marker Z-scores between all individual patient clusters in each meta-cluster was calculated as a metric for clustering optimization. Defining the features of each meta-cluster included markers with an absolute average Z-score greater than 5 and a significant change in expression in over 50% of the clusters within the meta-cluster. The proportion of marker change in each meta-cluster was calculated as the fraction of cells assigned to each meta-cluster, with significant changes accounting for the directionality. The MAST Z-scores for each marker were used as the input to meta-cluster equivalent sample clusters between samples using unsupervised phenograph clustering. The correlation of the Z-scores for all clusters assigned to a meta-cluster was calculated as a metric of how well the meta-clustering worked. Markers with an absolute value average Z-score greater than 5 and with a change in over 50% of the clusters within the meta-cluster were used as definitive markers for that meta-cluster. Additionally, the proportion of marker changes based on meta-cluster assignments was calculated by summing the number of cells assigned to each meta-cluster with the corresponding change. The number of phenotypic meta-clusters (N=13) was optimized using Rphenograph. Rphenograph produces a modularity value for how well the data are grouped together, based on various threshold values.

**References**

1. Smadbeck J, Peterson JF, Pearce KE, Pitel BA, Figueroa AL, Timm M*, et al.* Mate pair sequencing outperforms fluorescence in situ hybridization in the genomic characterization of multiple myeloma. Blood Cancer J. 2019;9:103.

2. Lin P, Owens R, Tricot G, Wilson CS. Flow cytometric immunophenotypic analysis of 306 cases of multiple myeloma. Am J Clin Pathol. 2004;121:482-8.
