# Supplementary material for: Mass Cytometry reveals unique phenotypic patterns associated with subclonal diversity and outcomes in multiple myeloma
Source: Blood Cancer J. 2023 May 22;13(1):84. doi: 10.1038/s41408-023-00851-5 (PMC10203138; doi:10.1038/s41408-023-00851-5)
Supplement: Supplementary file 4 — Supplementary Figures [file 41408_2023_851_MOESM4_ESM.pdf]

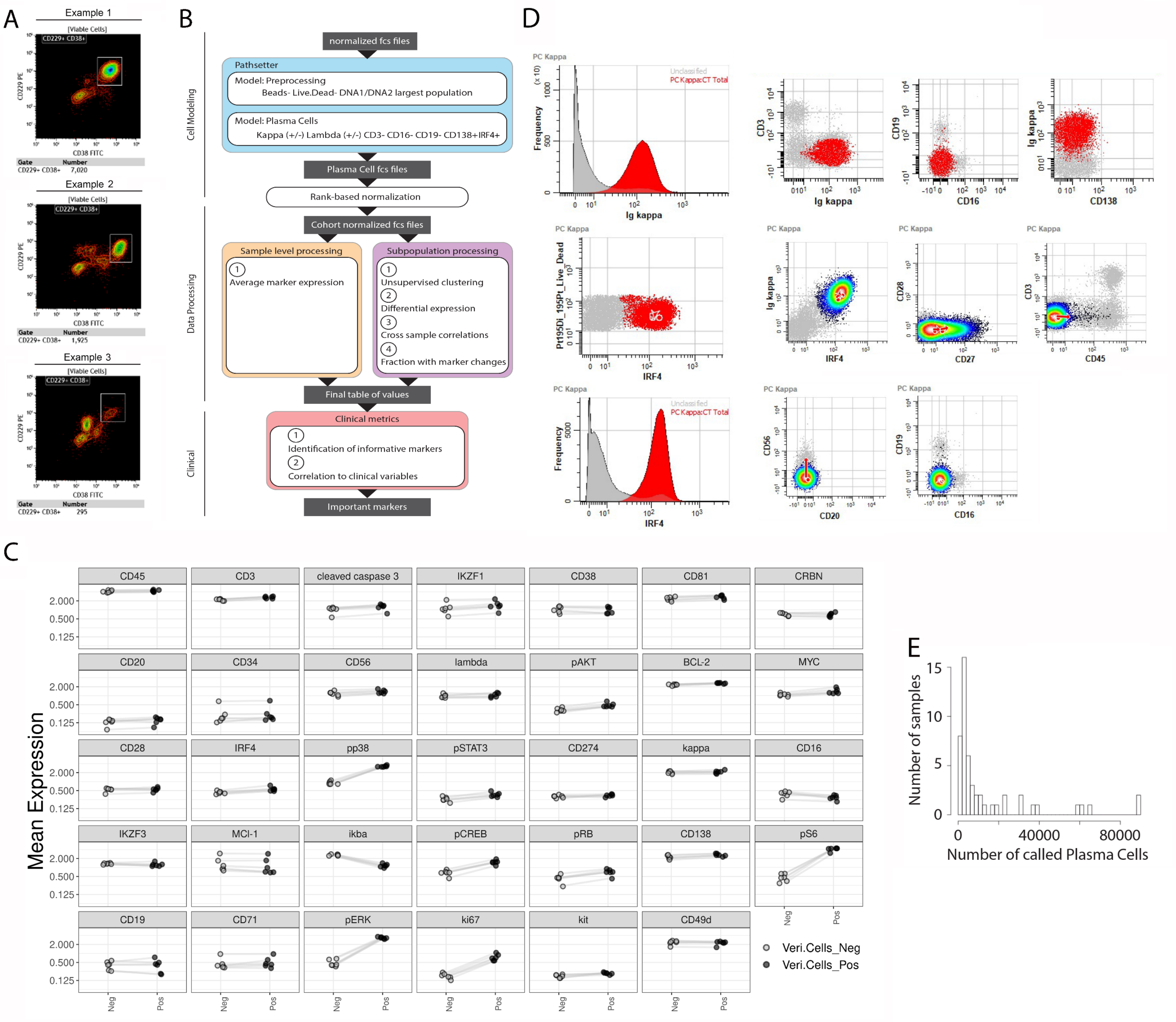

Supplementary Figure 1: Schematic of sample processing and gating approach. A) Prior to CyTOF analysis, three randomly selected cryopreserved whole BM samples obtained from the liquid nitrogen biobank were evaluated by flow cytometry to determine the abundance of viable PCs. From three example cases, the abundance of viable PCs that are CD38 and CD229 positive are indicated with a white box. B) Schematic of CyTOF sample processing utilizing the pathsetter software incorporating a preprocessing step of selecting live events and selection of PCs by CD3, CD16 and CD19 negativity and CD138, IRF4 and light chain positivity. Further data processing included average marker expression in each sample and subpopulation processing involving unsupervised clustering, differential expression, cross sample correlations and calculation of subpopulation fraction with marker changes. Final analysis included correlation with select clinical variables. C) Mean bulk expression of lyophilized Veri-Cells comparing untreated (Neg) and PMA and ionomycin stimulation (Pos). D) Pathsetter cell modeling example of a single whole BM samples demonstrating PC (marked in red or by the colored contour plots) positivity for kappa, IRF4 and negativity for CD3, CD16, CD19. Cryopreservation resulted in low CD138 positivity. The background grey histogram or dot plots represent the non-PC whole BM cells. E) Number of called PCs (Ig kappa+/-, Ig lambda-/+, CD3-, CD16-, CD19-, CD138+, IRF4+) from each patient used for the analyses.

Supplementary Figure 2

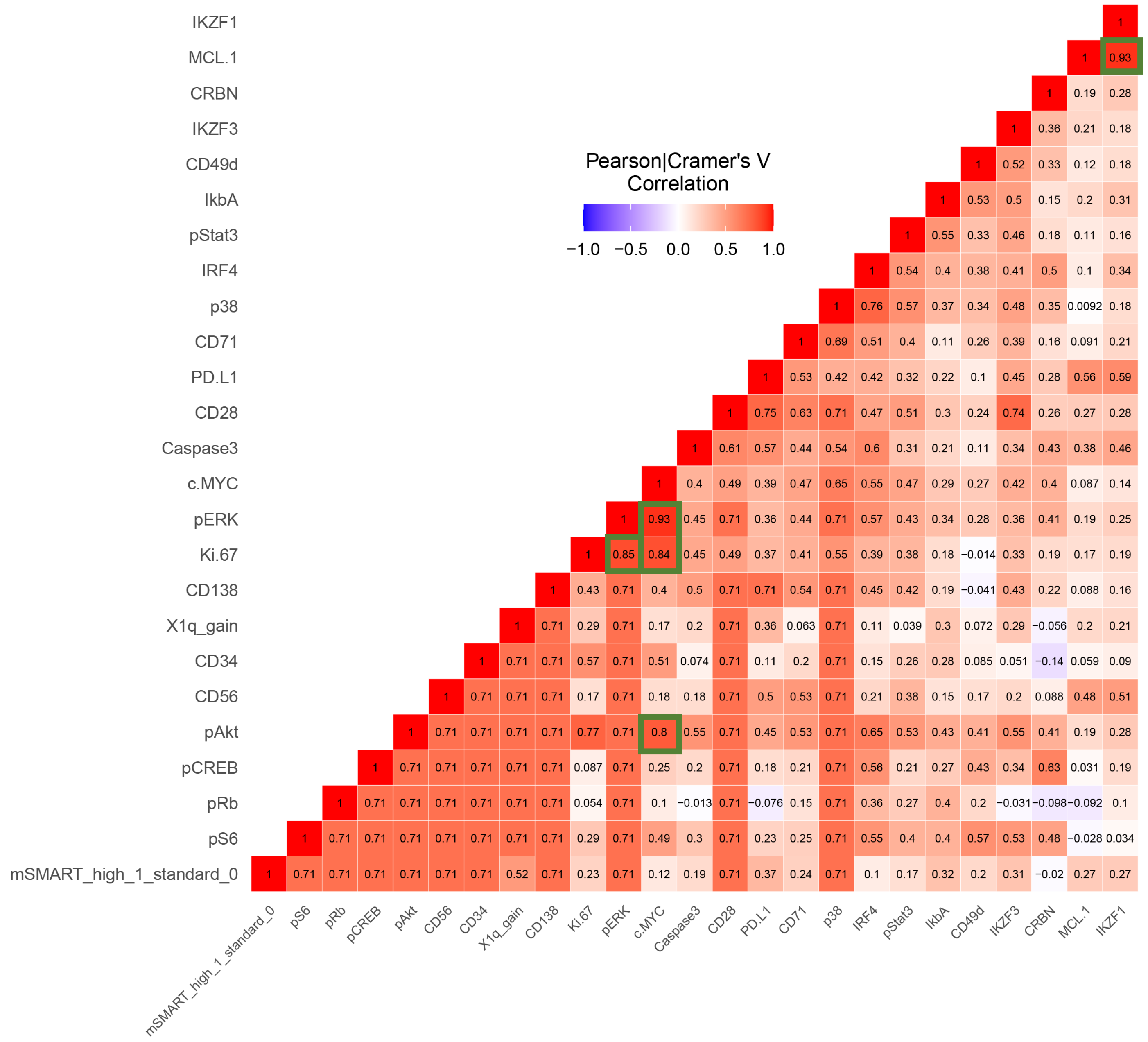

Supplementary Figure 2: Correlation coefficient between pairs of variables. Variables shown include mSMART (1 high, 0 standard), 1q gain (1 yes, 0 no) and the indicated protein markers. Correlation was calculated with Cramer's V method with signs (+/-) introduced from a Pearson's correlation test, which was used to estimate each correlation. Only variables with p<0.0001 are shown. Green box indicates correlations >0.8.

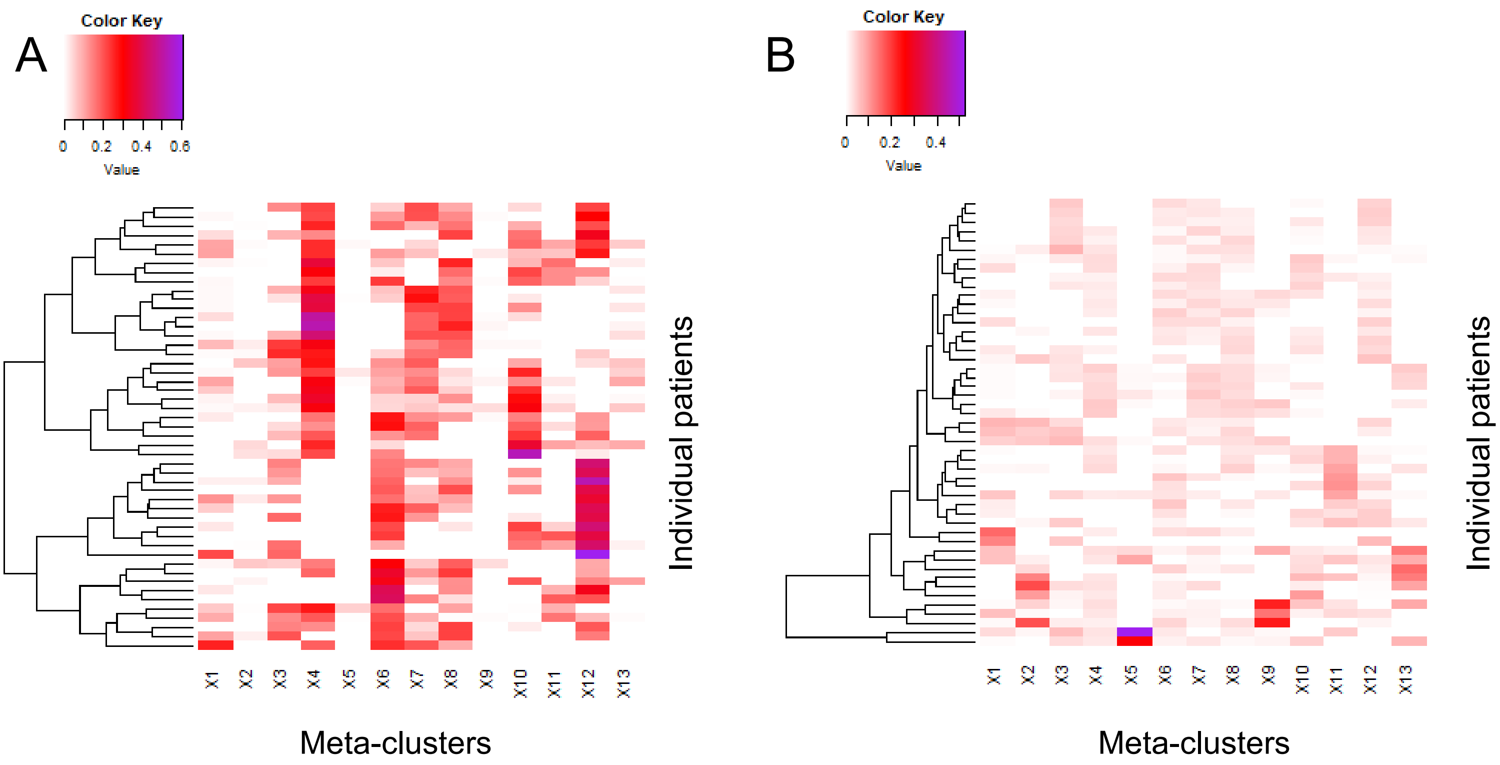

Supplementary Figure 3: Abundance of the 13 phenotypic meta-clusters within the 49 samples. A) Fraction of cells in each of the 49 patients belonging to each of the 13 phenotypic meta-clusters and B) fraction of cells in each phenotypic meta-cluster belonging to each of the 49 patients.

Supplementary Figure 4

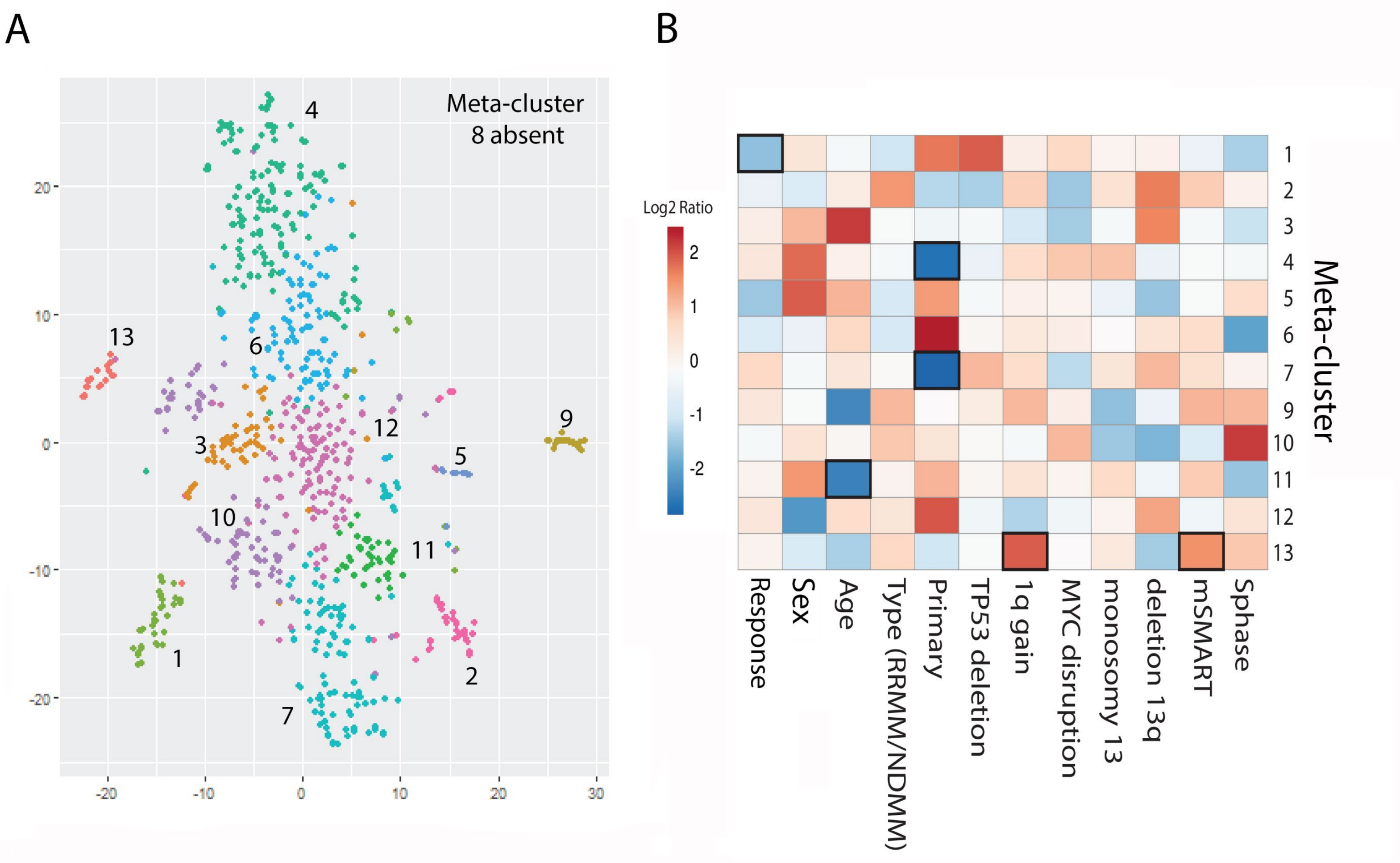

Meta-cluster	1	2	3	4	5	6	7	8	9	10	11	12	13
With CD38	3.5	0.7	6.6	19.1	0.2	14.8	10.3	11.1	0.4	10.6	3.6	17.4	1.5
Without CD38	3.4	1.3	6.6	24.4	0.2	15.8	11.0	0.0	0.5	12.5	4.9	17.9	1.5

Supplementary Figure 4: T-SNE plots of the 13 phenotypic meta-clusters and comparison with clinical metrics, patient demographics and tumor genetics. A) t-SNE of all unsupervised clusters between samples colored by the meta-cluster assignment with labelling of the significant markers in each meta-cluster with CD38 excluded. Meta-cluster 8 is absent. Bottom: the average abundance (percentage) of each meta-cluster in the analysis that included CD38 and excluded CD38. B) Heatmap from data excluding CD38 as a marker showing the log2 fold change in the proportion of cells within a sample belonging to each meta-cluster for different clinical and cohort metrics. Cohort metrics defining response to treatment (poor including PD vs good including PR and VGPR), sex, age, type (NDMM or RRMM), primary genetic abnormality, *TP53* deletion, *MYC* disruption, monosomy of 13, deletion 13q, mSMART score, and S-phase value. Significant ($P < 0.05$) changes are highlighted in a black box.
